# Global trends in systemic sclerosis-related mortality, 2001–2023: an epidemiological analysis using World Health Organization mortality data

**DOI:** 10.1007/s10067-026-07995-2

**Published:** 2026-03-06

**Authors:** Keith Pardillada Belangoy, Yoshito Nishimura, Ko Harada, Hideharu Hagiya, Quynh Thi Vu, Hanane Ouddoud, Judah Israel Ong Lescano, Michio Yamamoto, Tatsuaki Takeda, Hirofumi Hamano, Toshihiro Koyama, Yoshito Zamami

**Affiliations:** 1 Department of Health Data Science, Graduate School of Medicine, Dentistry, and Pharmaceutical Sciences, Okayama University, Okayama, 7008558 Japan; 2 Division of Haematology and Oncology, Mayo Clinic, Rochester, MN 55901 USA; 3 Brookdale Department of Geriatrics and Palliative Medicine, Icahn School of Medicine at Mount Sinai, New York, NY 100295674 USA; 4 Department of Infectious Diseases, Okayama University Hospital, Okayama, 7008558 Japan; 5 Graduate School of Human Sciences, The University of Osaka, Osaka, 5650871 Japan; 6 RIKEN Center for Advanced Intelligence Project, Tokyo, 1030027 Japan; 7 Data Science and AI Innovation Research Promotion Center, Shiga University, Shiga, 5228522 Japan; 8 Department of Education and Research Center for Clinical Pharmacy, Faculty of Pharmaceutical Sciences, Okayama University, Okayama, 7008558 Japan; 9 Department of Pharmacy, Okayama University Hospital, Okayama, 7008558 Japan; 10 Department of Pharmaceutics and Pharmaceutical Technology, Faculty of Pharmacy, Haiphong University of Medicine and Pharmacy, Haiphong, 180000 Vietnam; 11 Department of Pharmacy, University of San Carlos, Cebu City, 6000 Philippines

**Keywords:** Age-standardized mortality rate, Global health, Mortality trends, Sociodemographic index, Systemic sclerosis

## Abstract

**Objectives:**

This study aimed to evaluate the global trends in systemic sclerosis (SSc)-related mortality by age, sex, and geographic region. SSc is a multisystem autoimmune disease characterized by tissue fibrosis, vascular dysfunction, and multi-organ involvement, which is associated with a high mortality risk.

**Methods:**

Using the World Health Organization Mortality Database, we examined trends in SSc-related crude mortality rates (SSc-CRs) and age-standardized mortality rates (SSc-ASMR) per 1,000,000 population from 2001 to 2023. Locally weighted regression was applied to visualize long-term patterns, and Joinpoint regression was used to assess the national trends from 2010 to 2023.

**Results:**

Across 74 countries, 85,291 SSc-related deaths were reported, with 79.41% occurring in females. The SSc-CR steadily increased from 1.97 (95% confidence interval [CI]: 1.71–2.23) in 2001 to 2.34 (95% CI: 2.01–2.68) in 2023, while the SSc-ASMR decreased from 1.58 (95% CI: 1.42–1.74) to 1.29 (95% CI: 1.08–1.50), respectively. Regionally, mortality was the highest in the Western Pacific region and declined in the Americas and Europe, with temporal fluctuations. The SSc-ASMR was highest in countries with a middle sociodemographic index (SDI).

**Conclusions:**

While overall age-standardized mortality from SSc has declined in many regions, disparities persist. These results underscore the importance of sustaining research and enhancing disease awareness, as well as developing strategies to reduce mortality in high-risk populations and regions.

**Table:** 

**Key Points** • *First global analysis of mortality trends across 74 countries (2001–2023)* • *Age-standardized mortality declined globally, but crude mortality increased, with persistent female predominance* • *Findings highlight need for targeted strategies, early diagnosis, and improved care to reduce mortality*

**Supplementary Information:**

The online version contains supplementary material available at 10.1007/s10067-026-07995-2.

## Introduction

Systemic sclerosis (SSc), also known as scleroderma, is a rare and complex autoimmune disease affecting connective tissues [1]. The European Alliance of Associations for Rheumatology (EULAR) defines eight key clinical domains of SSc: Raynaud’s phenomenon, digital ulcers, pulmonary arterial hypertension, scleroderma renal crisis, skin fibrosis, interstitial lung disease, gastrointestinal manifestations, and musculoskeletal involvement [2]. Because of the persistent and progressing symptoms, SSc places a substantial burden on patients, impacting both quality of life and overall survival [1, 3].

Despite progress in understanding SSc and treating organ involvement, patients continue to experience significant morbidity and mortality [4]. SSc ranks highest in mortality among rheumatic diseases, according to the epidemiological data summarized by Volkmann et al. [1] Globally, an estimated 1.47 million individuals are affected, with a pooled prevalence of 18.87 per 100,000 persons [3]. SSc predominantly affects women, representing about 80% of cases among patients with autoimmune diseases [3, 5]. Both genetic susceptibility and environmental exposures are important determinants of SSc onset and outcome [6].

While SSc has been recognized as one of the leading causes of disability among systemic connective tissue disorders in the Global Burden of Disease Study 2019, SSc-specific mortality has not been reported, highlighting a critical gap. To our knowledge, this is the first study to report global SSc mortality trends using data from the World Health Organization (WHO) mortality database, providing new insights into the global burden of disease and associated temporal changes. This study aimed to evaluate changes in SSc mortality trends from 2001 to 2023, stratified by age, sex, and geographic region, to identify high-risk populations and inform public health strategies.

## Methods

### Data source

This observational study used mortality data from the WHO Mortality Database (last updated February 1, 2025), which provides detailed records of deaths by country, year, sex, and age group since 1950 [7]. SSc was defined as the underlying cause of death using the International Statistical Classification of Diseases and Related Health Problems, 10th Revision (ICD-10) code M34 [8]. The ICD-10 codes used to define systemic sclerosis are as follows: SSc (M34); progressive SSc (M34.0); CREST syndrome (M34.1), a combination of calcinosis, Raynaud phenomenon, esophageal dysfunction, sclerodactyly, and telangiectasia; SSc induced by drugs and chemicals (M34.2); other forms of SSc (M34.8), cases not classified under standard subtypes, often affecting internal organs (e.g., lungs, muscles, nerves); and SSc, unspecified (M34.9; SSc without specification of subtype or organ involvement) [8].

### Statistical analyses and data processing

The statistical analysis workflow is illustrated in Supplementary Fig. S1. We analyzed SSc-related mortality data from 2001 to 2023, stratified by 5-year age groups (from 0 to 85 years or older). Countries with no reported SSc deaths were treated as true zeros, as in the previous study [9]. Population denominators were obtained from the United Nations World Population Prospects 2024 [10]. For each year from 2001 to 2023, age-specific mortality rates were calculated for each age group. Crude mortality rates (CRs) were calculated by dividing the total number of deaths by the total population. The CRs were then age-standardized using the New WHO World Standard Population Distribution [11] and reported as deaths per 1,000,000 population.

A long-term global trend in SSc-related mortality from 2001 to 2023 was modeled using locally weighted regression (LOESS), weighted by each country’s population, with 95% confidence intervals (CIs) calculated [12]. The LOESS-smoothed mortality trends were then stratified by WHO regions [13], and further stratified using the sociodemographic index (SDI) quintiles, grouping countries into high, high-middle, and middle-SDI categories [14].

Joinpoint regression software (version 5.4.0.0 April, 2025; Statistical Research and Applications Branch, National Cancer Institute) was used to analyze mortality trends from 2010 to 2023 for each country, calculating the average annual percentage change (AAPC) in mortality rates and its 95% CI [15]. Statistical significance for trends was defined as *p* < 0.05 with a 95% CI for the AAPC that did not include zero. Data were processed and aggregated in Microsoft Access 2013 (Microsoft Corporation, Redmon, WA, USA). Statistical analyses were performed using R software (version 2025.05.0 [Build 496]).

### Inclusion and exclusion criteria

Inclusion of countries depended on the quality of their vital registration data, assessed via usability metric based on the latest score from 2008 to 2019: scores of 80% or higher indicated high quality, 60–79% as medium quality, and below 60% as low quality [16]. We analyzed data only from countries classified as having medium to high data quality. Furthermore, for the LOESS analysis, only countries with at least 11 years of data between 2001 and 2023 were included to ensure sufficient variability and reliable smoothed trends analysis. For Joinpoint regression analysis, countries were excluded if they reported zero SSc deaths in any study year or had fewer than 7 years of data from 2010 to 2023.

This study was performed according to the REporting of studies Conducted using the Observational Routinely collected health Data (RECORD) Statement [17].

### Ethical approval

This study used publicly available data, and informed consent was not required as it involved retrospective analysis of routinely collected data.

### Role of funding source

The funders had no role in the study design, data collection, data analysis, data interpretation, or decision to submit the manuscript for publication.

## Results

Overall, 85,291 SSc-related deaths were reported across 74 countries included in the LOESS analysis from 2001 to 2023. Eligible countries, grouped by WHO region and SDI classification, are listed in Supplementary Table S1. For the Joinpoint regression analysis, 47 countries met the inclusion criteria (Supplementary Table S2), and AAPCs were calculated for 2010–2023.

**Fig. 1:**
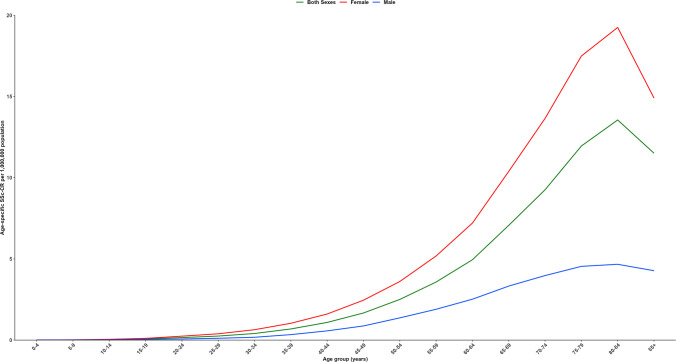
Age-specific SSc-CR per 1,000,000 population, by sex, 2001–2023. SSc-CR = systemic sclerosis crude rate

The leading causes of SSc mortality were other forms of SSc (ICD-10: M34.8, 48.64%) and unspecified SSc (M34.9, 35.43%) (Supplementary Table S3). SSc-related deaths occurred predominantly in women (79.40%) and age-specific SSc-CRs increased with age, rising from 1.09 per 1,000,000 population in the 40–44-year age group to a peak of 13.55 per 1,000,000 people at 80–84 years, for both sexes, followed by a decline to 11.51 per 1,000,000 population in those aged above 85 years (Fig. 1 and Supplementary Table S4).

**Fig. 2:**
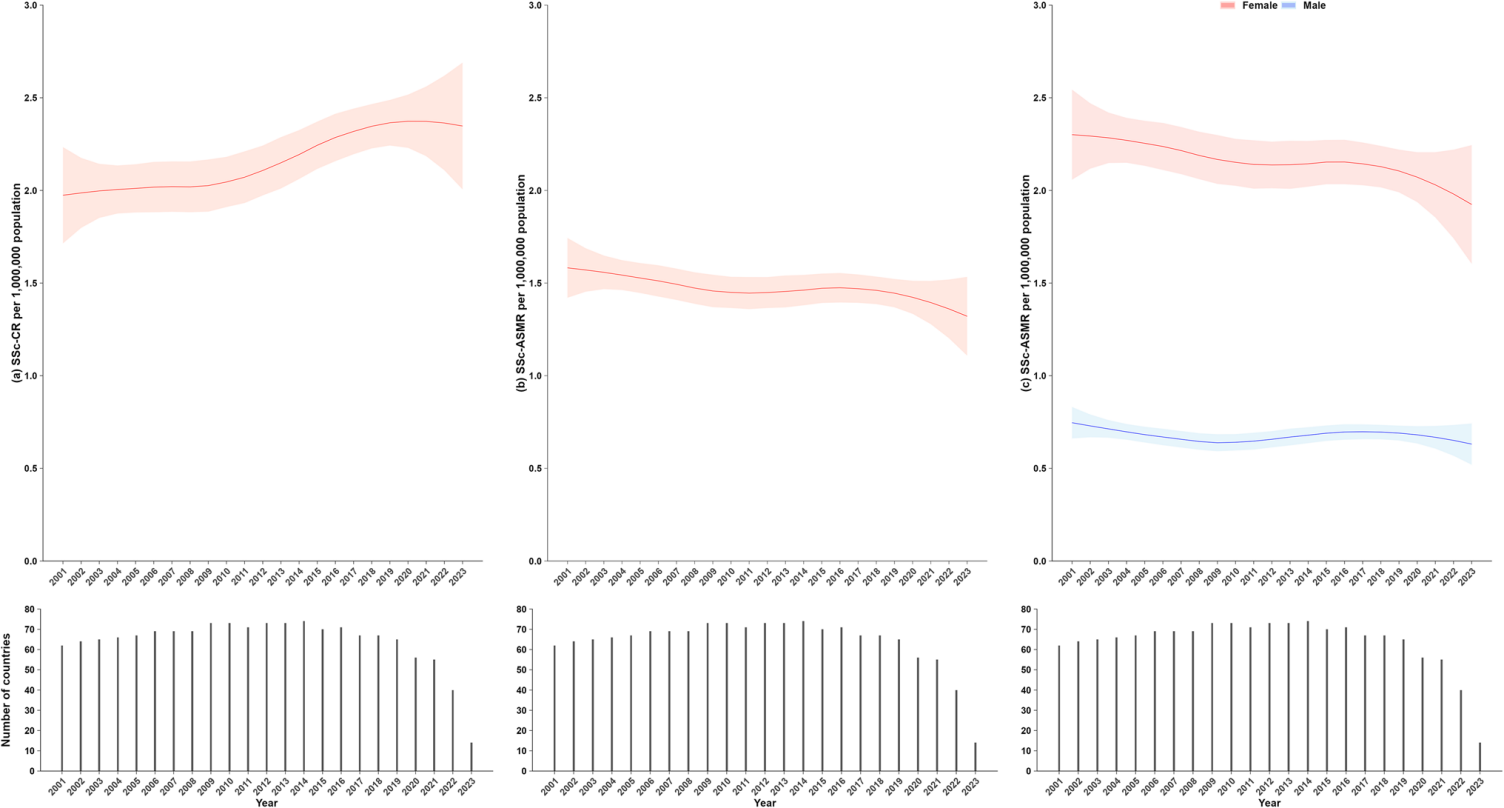
Trends in SSc-CR and SSc-ASMR per 1,000,000 population across 74 countries, 2001–2023. **a** SSc-CR; **b** ASSc-ASMR; **c** SSc-ASMR by sex group. The locally weighted regression (LOESS) rates (red lines) with 95% confidence intervals (light red shadows) are shown in panels A and B. The LOESS rates for males (blue line) and females (red line) with 95% confidence intervals (light blue and light red shadows, respectively) are shown in panel C. SSc-CR = systemic sclerosis crude rate; SSc-ASMR = systemic sclerosis age-standardized mortality rate

The SSc-CR for the 74 countries, showed a consistent upward trend, increasing from 1.97 (95% CI: 1.71–2.23) per 1,000,000 people in 2001 to 2.34 (95% CI: 2.01–2.68) per 1,000,000 population in 2023. Conversely, the age-standardized mortality rate (SSc-ASMR), per 1,000,000 population, declined from 1.58 (95% CI: 1.42–1.74) in 2001 to 1.29 (95% CI: 1.08–1.50) in 2023. Global trends in SSc-CR and SSc-ASMR are presented in Fig. 2 and Supplementary Table S5, and detailed country-specific SSc-ASMR data are provided in Supplementary Table S6. The LOESS estimates of SSc-ASMR, stratified by sex, declined over the past 23 years (Supplementary Table S7). The SSc-ASMR in men showed a slight decline from 0.75 (95% CI: 0.66–0.83) in 2001 to 0.62 (95% CI: 0.51–0.73) in 2023. The SSc-ASMR remained higher in females than in males throughout the study period; however, results showed an evident decrease from 2.30 (95% CI: 2.06–2.54) in 2001 to 1.87 (95% CI: 1.56–2.19) in 2023.

**Fig. 3:**
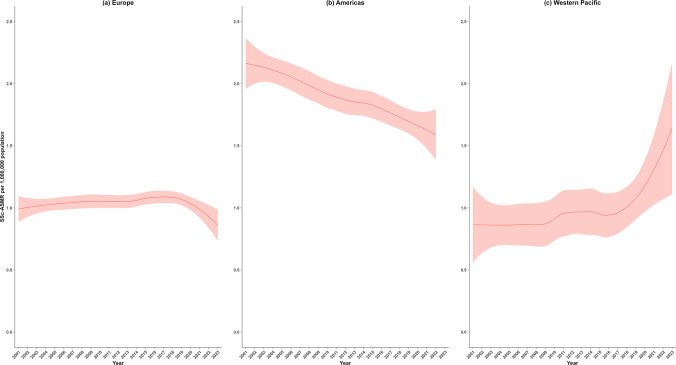
Trends in SSc-ASMR by WHO region, 2001–2023. The locally weighted regression (LOESS) rates (red line) with 95% confidence intervals (light red shadow) during 2001–2023 are shown for (**a**) Europe; (**b**) Americas; and (**c**) Western Pacific. SSc-ASMR = systemic sclerosis age-standardized mortality rate

Figure 3 shows the SSc-ASMR (from 2001 to 2023) according to WHO regional classification, with detailed numbers listed in Supplementary Table S8. The LOESS estimates of SSc-ASMR showed a two-fold increase in Western Pacific from 0.86 (95% CI: 0.56–1.17) in 2001 to 1.64 (95% CI: 1.11–2.17) in 2023. In contrast, a consistent downward trend was observed in the Americas, from 2.16 (95% CI: 1.96–2.37) in 2001 to 1.59 (95% CI: 1.39–1.79) in 2022, with no available data for 2023. The SSc-ASMR in Europe showed fluctuations over the study period and declined from 0.99 (95% CI: 0.89–1.10) in 2001 to 0.84 (95% CI: 0.72–0.96) in 2023.

**Fig. 4:**
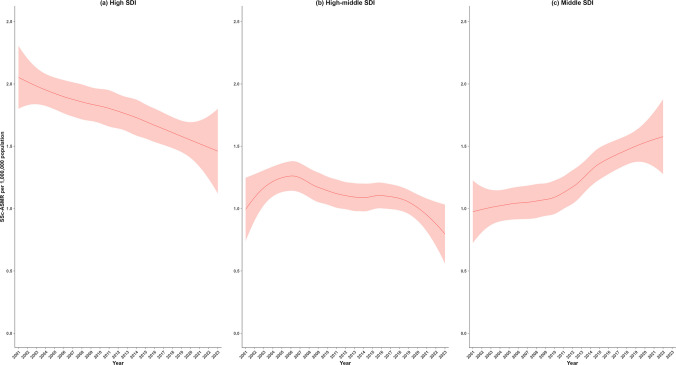
Trends in SSc-ASMR by SDI group, 2001–2023. The locally weighted regression (LOESS) rates (red line) with 95% confidence intervals (light red shadow) for 2001–2023 are shown for (**a**) high SDI; (**b**) high-middle SDI; and (**c**) Middle SDI. SSc-ASMR = systemic sclerosis age-standardized mortality rate; SSc = systemic sclerosis; SDI = sociodemographic index

Figure 4 shows the LOESS estimates for SSc-ASMR, stratified by SDI, with detailed data provided in Supplementary Table S9. The middle-SDI group had the highest SSc-ASMR, increasing from 0.98 (95% CI: 0.72–1.23) in 2001 to 1.58 (95% CI: 1.28–1.88) in 2022 with no available data for 2023. While a sharp decline from 2.05 (95% CI: 1.80–2.30) in 2001 to 1.39 (95% CI: 1.06–1.72) in 2023 was observed in the high SDI group and high-middle-SDI group from 0.99 (95% CI: 0.74–1.25) in 2001 and dropped to 0.80 (95% CI: 0.56–1.03) in 2023.

**Fig. 5:**
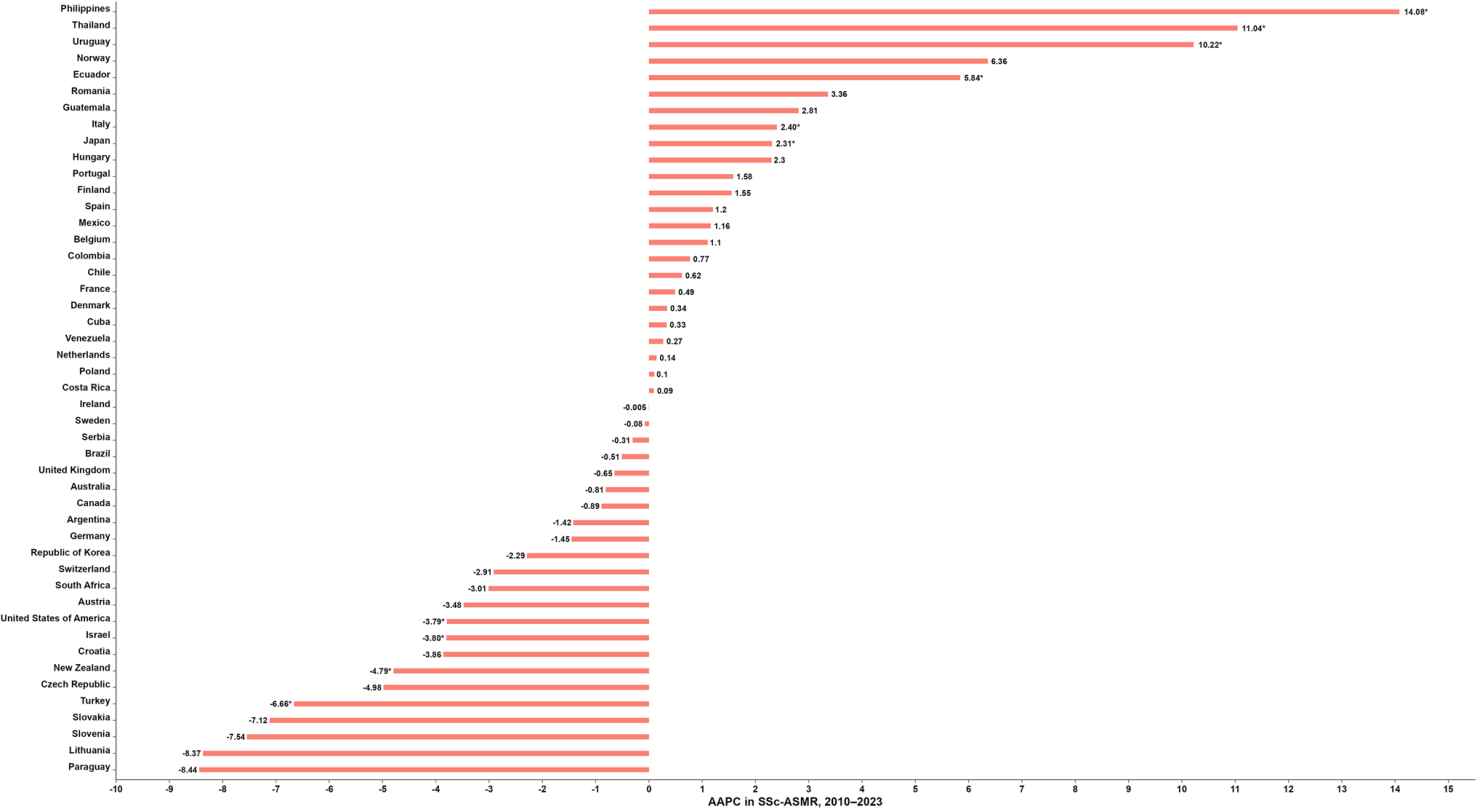
AAPC in SSc-ASMR, 2010–2023. *AAPC was considered statistically significant if *p* < 0.05 and the 95% CI did not include zero. AAPC = average annual percentage change; SSc-ASMR = systemic sclerosis age-standardized mortality rate

The AAPC in SSc-ASMR between 2010 and 2023, by country, is shown in Fig. 5 and Supplementary Table S10. Among 47 countries, most showed stable trends, with highest AAPCs observed in the Philippines (+14.08%, 95% CI: 5.54–35.81) and Thailand (+11.04%, 95% CI: 7.59–16.73), while Paraguay (−8.44%, 95% CI: −19.51–5.08) and Lithuania (−8.37%, 95% CI: −18.28–2.65) showed greatest declines.

## Discussion

Our study is the first to clarify global SSc-related mortality from 2001 to 2023. The results showed an increasing SSc-CR but declining SSc-ASMR. This may have been driven by population ageing and longer survival with SSc, while the decline in ASMR is consistent with reduction in age-specific mortality risk associated with earlier diagnosis and improved disease management. However, persistent disparities were observed across age, sex, and region, particularly with the highest mortality observed in the Western Pacific region and in the middle-SDI countries.

Consistent with previously reported patterns [4, 5, 18, 19], our analysis showed that mortality rates increased with age and were nearly four times higher in females than in males. These findings are consistent with the findings of studies from France [18] and the UK [19]. The higher mortality rates in females are likely driven by the greater prevalence of disease [5, 6]. In Mexico, males also showed increasing AAPC trends, suggesting that the burden is rising across both sexes [20]. These trends may reflect later diagnosis and more severe disease expression, including internal organ involvement, partially contributing to the observed mortality patterns [4, 5].

The rising mortality in the Western Pacific region, observed in our study, may reflect geographical differences shaped by genetic factors [6, 21, 22]. Evidence suggests that human leukocyte antigen (HLA) plays a role in SSc development, with susceptibility varying across populations [6]. Future studies to associate epidemiology data with population-wide genomic analyses are warranted to verify the hypothesis. The observed trends may partly reflect the reported high prevalence of certain SSc subtypes in Japan, Korea, and Singapore [23]. In addition, multi-racial cohort studies have reported varying organ involvement, with interstitial lung disease being more common in patients from Korea and Japan and scleroderma renal crisis being more frequently observed in Australia, the U.S., and Europe [1, 23]. Together, these observations suggest that racial differences and varying intensity of clinical evaluation contribute to SSc-related mortality across populations.

The declining mortality trends observed in high- and high-middle-SDI regions appear to reflect improved disease recognition; the use of enhanced classification has emphasized the relevance of early clinical evaluation, as these measures are linked with nearly 80% probability of progression to definite SSc [1, 24]. These classification approaches have been shown to support diagnostic confirmation and earlier recognition of organ involvement, thereby informing timely clinical management before irreversible organ damage develops [1, 20, 24]. Overall, improvement in diagnosis and classification may have led to long-term decline in morbidity and mortality. Greater physician awareness, earlier disease detection, and broader use of disease-modifying therapies likely further contribute to better outcomes in developed countries [1, 25], as exemplified by the U.S. [25] and Canada [26].

In contrast, increasing mortality in the middle-SDI regions may appear to be driven, in part, by limited disease awareness, delayed diagnosis, and barriers to advanced treatment [21]. This has been observed in Mexico [20], whereas in Thailand, genetic susceptibility, environmental factors, and a high reported prevalence of SSc in the country (24.4 per 100,000 population in 2017) likely also contribute [27]. In addition, varying environmental exposures, such as silica and organic solvents, have been noted as possible contributors to SSc development, warranting additional investigation [6]. These trends may also reflect disparities in access to rheumatology services, with regional shortfalls highlighting uneven healthcare capacity and specialist availability [28]. Differences in healthcare access, treatment availability, and associated costs may impact disease progression in SSc and influence related mortality [23]. Recognizing these disparities, along with disease characteristics and treatment approaches, could help guide future strategies to improve management of SSc, supporting better outcomes.

This study has some limitations. First, the accuracy of mortality rates depends on the quality of death certification and the recorded causes of death. Second, most included countries were from Europe and the Americas, where data quality is generally high, and fewer countries from Southeast Asia, Africa, and the Eastern Mediterranean were included due to lower-quality or incomplete data. Lastly, misclassification of SSc during diagnosis and death reporting may have influenced the accuracy of mortality estimates, potentially leading to underestimation or overestimation of mortality rates in some regions. LOESS-smoothed mortality rates were limited to 74 countries with available data included in this study.

## Conclusion

This study demonstrated the global trends of SSc-related mortality over the past two decades, particularly highlighting the persistence of sex disparities and the rising trends observed in countries within the Western Pacific region and those with middle-SDI levels. These findings emphasized the need to improve disease awareness and access to expert centers in regions with increasing mortality. Future studies should integrate clinical, genetic, environmental, and sociodemographic data to elucidate regional disparities in SSc outcomes.

## Supplementary information

ESM 1 Fig. S1 Statistical Analysis Workflow. Table S1 Breakdown list of countries included in the LOESS analysis (74 countries). Table S2 Breakdown list of countries included in the Joinpoint analysis (47 countries). Table S3 Death percentages by ICD-10 code from 2001 to 2023 across 74 countries included in the LOESS analysis. Table S4 Age-specific SSc-CR mortality. Table S5 SSc-CR and SSc-ASMR across 74 countries, 2001–2023. Table S6 Country-specific SSc-ASMR, 2001–2023. Table S7 SSc-ASMR of male and female population across 74 countries. Table S8 SSc-ASMR across 74 countries sorted by regions. Table S9 SSc-ASMR across 74 countries sorted by SDI group. Table S10 AAPC SSc-ASMR, 2010–2023 (PDF 580 kb)

ESM 2 (DOCX 34 kb)

## Data Availability

All data used in this study are from the World Health Organization (WHO) Mortality Database and are publicly accessible online.
