# Supplementary material for: Global trends in systemic sclerosis-related mortality, 2001–2023: an epidemiological analysis using World Health Organization mortality data
Source: Clin Rheumatol. 2026 Mar 6;45(5):2741–8. doi: 10.1007/s10067-026-07995-2 (PMC13068748; doi:10.1007/s10067-026-07995-2)
Supplement: Supplementary file 1 — Fig. S1 Statistical Analysis Workflow. Table S1 Breakdown list of countries included in the LOESS analysis (74 countries). Table S2 Breakdown list of countries included in the Joinpoint analysis (47 countries). Table S3 Death percentages by ICD-10 code from 2001 to 2023 across 74 countries included in the LOESS analysis. Table S4 Age-specific SSc-CR mortality. Table S5 SSc-CR and SSc-ASMR across 74 countries, 2001–2023. Table S6 Country-specific SSc-ASMR, 2001–2023. Table S7 SSc-ASMR of male and female population across 74 countries. Table S8 SSc-ASMR across 74 countries sorted by regions. Table S9 SSc-ASMR across 74 countries sorted by SDI group. Table S10 AAPC SSc-ASMR, 2010–2023 (PDF 580 kb) [file 10067_2026_7995_MOESM1_ESM.pdf]

Supplementary Materials

Table of Contents

Supplementary Figure

Figure S1 Statistical Analysis Workflow..1

Supplementary Tables

Table S1 Breakdown list of countries included in the LOESS analysis (74 countries) 2

Table S2 Breakdown list of countries included in the Joinpoint analysis (47 countries).........2

Table S3 Death percentages by ICD-10 code from 2001 to 2023 across 74 countries included in the LOESS analysis 3

Table S4 Age-specific SSc-CR mortality 3

Table S5 SSc-CR and SSc-ASMR across 74 countries, 2001–2023 4

Table S6 Country-specific SSc-ASMR, 2001–2023 4

Table S7 SSc-ASMR of male and female population across 74 countries 7

Table S8 SSc-ASMR across 74 countries sorted by regions 7

Table S9 SSc-ASMR across 74 countries sorted by SDI group 8

Table S10 Trends in AAPC SSc-ASMR during 2010–2023 8

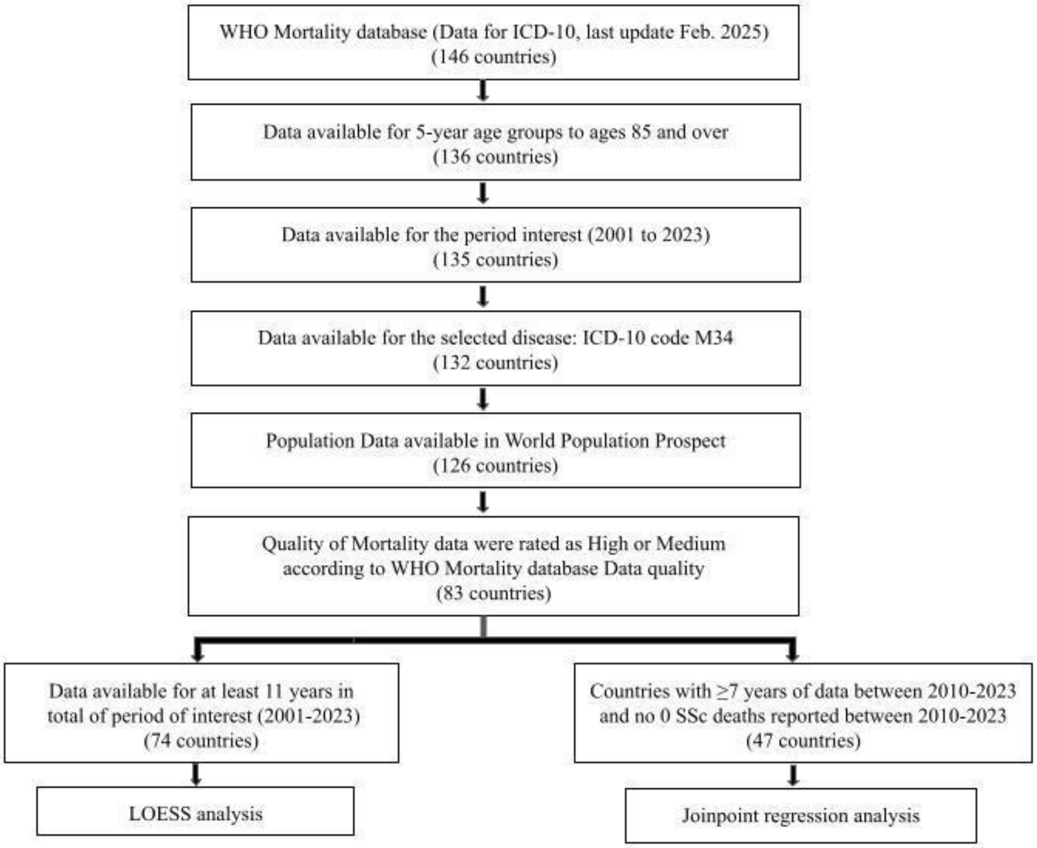

Figure S1: Statistical analysis workflow (Adapted from Hagiya H, Harada K, Nishimura Y, et al. Global trends in mortality related to pulmonary embolism: An epidemiological analysis of data from the World Health Organization mortality database from 2001 to 2023. *Clinical Medicine* 2025; **86**:103389**)**

Table S1. Breakdown list of countries included in the LOESS analysis (74 countries)

By the socio-demographic index (SDI)

**SDI high**<br>30 countries	Australia, Austria, Belgium, Canada, Cyprus, Czech Republic, Denmark, Estonia, Finland, France, Germany, Iceland, Ireland, Japan, Kuwait, Latvia, Lithuania, Luxembourg, Netherlands, New Zealand, Norway, Poland, Republic of Korea, Singapore, Slovakia, Slovenia, Sweden, Switzerland, United Kingdom, United States of America
**SDI High-middle**<br>20 countries	Antigua and Barbuda, Argentina, Bahamas, Chile, Croatia, Dominica, Georgia, Hungary, Israel, Italy, Malta, Mauritius, Portugal, Republic of Moldova, Romania, Saint Kitts and Nevis, Serbia, Spain, Turkey, Uruguay
**SDI Middle**<br>19 countries	Armenia, Brazil, Colombia, Costa Rica, Cuba, Ecuador, Grenada, Guyana, Jamaica, Mexico, Panama, Paraguay, Philippines, Saint Lucia, Saint Vincent and Grenadines, South Africa, Suriname, Thailand, Uzbekistan
**SDI Low-middle**<br>5 countries	Belize, Guatemala, Kyrgyzstan, Nicaragua, Venezuela

By Region

**Americas**<br>27 countries	Antigua and Barbuda, Argentina, Bahamas, Belize, Brazil, Canada, Chile, Colombia, Costa Rica, Cuba, Dominica, Ecuador, Grenada, Guatemala, Guyana, Jamaica, Mexico, Nicaragua, Panama, Paraguay, Saint Kitts and Nevis, Saint Lucia, Saint Vincent and Grenadines, Suriname, United States of America, Uruguay, Venezuela
**Europe**<br>37 countries	Armenia, Austria, Belgium, Croatia, Cyprus, Czech Republic, Denmark, Estonia, Finland, France, Georgia, Germany, Hungary, Iceland, Ireland, Israel, Italy, Kyrgyzstan, Latvia, Lithuania, Luxembourg, Malta, Netherlands, Norway, Poland, Portugal, Republic of Moldova, Romania, Serbia, Slovakia, Slovenia, Spain, Sweden, Switzerland, Turkey, United Kingdom, Uzbekistan
**Western Pacific**<br>6 countries	Australia, Japan, New Zealand, Philippines, Republic of Korea, Singapore
**Others**<br>4 countries	Kuwait, Mauritius, South Africa, Thailand

Table S2. Breakdown list of countries included in the Joinpoint analysis (47 countries)

By the socio-demographic index (SDI)

**SDI high**<br>23 countries	Australia, Austria, Belgium, Canada, Czech Republic, Denmark, Finland, France, Germany, Ireland, Japan, Lithuania, Netherlands, New Zealand, Norway, Poland, Slovakia, Slovenia, Republic of Korea, Sweden, Switzerland, United Kingdom, United States of America
**SDI High-middle**<br>12 countries	Argentina, Chile, Croatia, Hungary, Israel, Italy, Portugal, Romania, Serbia, Spain, Turkey, Uruguay
**SDI Middle**<br>10 countries	Brazil, Colombia, Costa Rica, Cuba, Ecuador, Mexico, Paraguay, Philippines, South Africa, Thailand
**SDI Low-middle**<br>2 countries	Guatemala, Venezuela

By Region

**Americas**<br>14 countries	Argentina, Brazil, Canada, Chile, Colombia, Costa Rica, Cuba, Ecuador, Guatemala, Mexico, Paraguay, United States of America, Uruguay, Venezuela

**Europe**<br>26 countries	Austria, Belgium, Croatia, Czech Republic, Denmark, Finland, France, Germany, Hungary, Ireland, Israel, Italy, Lithuania, Netherlands, Norway, Poland, Portugal, Romania, Serbia, Slovakia, Slovenia, Spain, Sweden, Switzerland, Turkey, United Kingdom
**Western Pacific**<br>5 countries	Australia, Japan, New Zealand, Philippines, Republic of Korea
**Others**<br>2 countries	South Africa, Thailand

Table S3: Death percentages by ICD-10 code from 2001 to 2023 across 74 countries included in the LOESS analysis

ICD-10 codes	Description	Percentage (%)	(case/case total)
M34	Systemic Sclerosis	3.75	3201 / 85291
M34.0	Progressive systemic sclerosis	4.81	4104 / 85291
M34.1	CR(E)ST syndrome	7.32	6247 / 85921
M34.2	Systemic Sclerosis induced by drugs and chemicals	0.04	35 / 85291
M34.8	Other forms of Systemic Sclerosis	48.64	41482 / 85291
M34.9	Systemic Sclerosis, unspecified	35.43	30222 / 85291

ICD-10 = International Statistical Classification of Diseases and Related Health Problems, 10th Revision; LOESS = locally weighted regression

Table S4: Age-specific SSc-CR

Age group	Male	Female	Both Sex
0-4	0.01	0.01	0.01
5-9	0.01	0.02	0.01
10-14	0.01	0.05	0.03
15-19	0.04	0.11	0.07
20-24	0.07	0.24	0.15
25-29	0.11	0.39	0.25
30-34	0.17	0.64	0.41
35-39	0.34	1.04	0.69
40-44	0.57	1.61	1.09
45-49	0.87	2.46	1.67
50-54	1.37	3.61	2.50
55-59	1.90	5.18	3.58
60-64	2.52	7.21	4.95
65-69	3.33	10.39	7.07
70-74	3.97	13.65	9.26
75-79	4.54	17.49	11.95
80-84	4.67	19.25	13.55
85+	4.27	14.90	11.51

Data are number of deaths per 1,000,000 population; SSc-CR = systemic sclerosis crude rate

Table S5: SSc-CR and SSc-ASMR across 74 countries, 2001–2023

SSc-CR

Year	2001	2002	2003	2004	2005	2006	2007	2008	2009	2010	2011	2012	2013	2014	2015	2016	2017	2018	2019	2020	2021	2022	2023
**LOESS smoothed rate**	1.97	1.99	2.00	2.01	2.01	2.02	2.02	2.02	2.03	2.05	2.07	2.11	2.15	2.19	2.24	2.29	2.32	2.35	2.36	2.37	2.37	2.36	2.34
**Upper 95% CI**	2.23	2.18	2.14	2.13	2.14	2.15	2.16	2.16	2.17	2.18	2.21	2.24	2.29	2.33	2.37	2.41	2.44	2.46	2.49	2.51	2.55	2.61	2.68
**Lower 95% CI**	1.71	1.80	1.85	1.88	1.88	1.88	1.88	1.88	1.89	1.91	1.93	1.97	2.01	2.06	2.12	2.16	2.20	2.23	2.24	2.23	2.19	2.11	2.01

SSc-ASMR

Year	2001	2002	2003	2004	2005	2006	2007	2008	2009	2010	2011	2012	2013	2014	2015	2016	2017	2018	2019	2020	2021	2022	2023
**LOESS smoothed rate**	1.58	1.57	1.56	1.54	1.53	1.51	1.49	1.47	1.46	1.45	1.45	1.45	1.45	1.46	1.47	1.48	1.47	1.46	1.44	1.41	1.38	1.34	1.29
**Upper 95% CI**	1.74	1.69	1.65	1.62	1.61	1.60	1.58	1.56	1.54	1.53	1.53	1.53	1.54	1.54	1.55	1.56	1.55	1.53	1.51	1.50	1.49	1.49	1.50
**Lower 95% CI**	1.42	1.45	1.47	1.46	1.45	1.43	1.41	1.39	1.37	1.37	1.36	1.37	1.37	1.38	1.39	1.40	1.39	1.38	1.36	1.32	1.26	1.18	1.08

Data are number of deaths per 1,000,000 population. SSc-CR = systemic sclerosis crude rate; SSc-ASMR = systemic sclerosis age-standardized mortality rate; LOESS = locally weighted regression; CI = confidence interval; NA = not available.

Table S6: Country-specific SSc-ASMR, 2001–2023

Country	2001	2002	2003	2004	2005	2006	2007	2008	2009	2010	2011	2012	2013	2014	2015	2016	2017	2018	2019	2020	2021	2022	2023
Antigua and Barbuda	0.00	0.00	0.00	0.00	0.00	0.00	15.30	0.00	0.00	0.00	0.00	0.00	0.00	0.00	0.00	0.00	0.00	0.00	0.00	0.00	0.00		
Argentina	0.74	1.08	1.62	1.47	1.12	1.12	1.36	1.28	1.14	1.20	1.30	1.11	1.57	1.53	1.61	1.34	1.06	1.07	1.29	1.12	1.17	1.21	
Armenia						0.73	0.93	0.25	1.09	0.62	0.48	0.67	0.74	0.98	0.37	0.55	0.00	0.43	0.71	0.22	0.41	0.43	
Australia	3.11	3.29	2.80	2.93		3.28	2.70	2.47	3.61	3.29	2.78	3.01	2.73	2.74	2.88	2.64	2.48	2.81	2.74	2.49	2.81	2.85	2.67
Austria		0.45	0.57	0.83	0.38	0.49	0.91	0.92	1.15	1.43	1.19	1.33	0.83	0.70	0.71	0.85	1.07	0.85	1.06	0.56	0.85	0.71	0.93
Bahamas	3.05	6.04	3.48	0.00	0.00	0.00	2.86	3.06	7.44	0.00	2.33	2.55	0.00	2.16	6.46								
Belgium	1.40	1.15	1.20	0.91	1.11	0.87	0.77	1.22	1.29	0.93	1.46	1.20	0.83	1.24	1.18	1.24	1.57	1.42	1.18	1.11	1.24		
Belize	0.00	0.00	0.00	0.00	6.18	0.00	0.00	0.00	0.00	0.00	0.00	2.76	0.00	0.00	0.00	0.00							
Brazil	0.72	0.95	1.00	1.00	1.01	1.04	0.93	1.10	0.95	0.92	0.98	1.00	1.03	1.04	1.17	1.14	1.09	1.15	1.05	0.88	0.90		
Canada	3.35	2.97	3.32	2.72	2.87	3.37	2.82	2.64	2.56	2.47	2.50	2.44	2.56	2.78	2.35	2.71	2.81	2.47	2.38	2.09	2.61	2.10	
Chile	1.91	2.14	2.72	2.65	2.09	1.71	2.79	1.78	1.58	1.30	1.38	1.80	1.97	1.25	1.26	1.53	1.72	1.19	1.88	1.68	1.48		
Colombia	1.23	1.08	1.55	1.10	1.21	1.11	1.14	1.71	1.28	1.29	1.64	1.69	1.90	1.69	2.10	2.18	2.03	2.24	2.47	1.86	1.68		
Costa Rica	4.06	1.40	3.54	1.78	2.78	3.55	2.39	2.42	2.57	2.28	1.33	2.19	2.27	2.76	1.18	2.19	1.84	1.77	3.09	1.45	2.02	2.14	
Croatia	0.56	0.43	0.51	0.50	0.37	2.08	0.75	0.81	0.49	0.65	1.58	1.09	0.27	0.95	1.22	1.12	0.40	0.57	1.08	0.46	0.90		

Cuba	1.86	1.08	1.63	0.81	1.30	1.62	1.31	0.57	1.20	1.39	1.12	2.01	1.01	0.59	1.46	1.17	1.12	1.59	1.84	1.03	1.40		
Cyprus				0.91	0.86	0.00	2.22	2.31	0.55	0.77	3.56	0.00	3.26	2.20	0.66	0.82	2.17	2.56	2.10	1.43	1.91	1.28	
Czech Republic	0.18	0.06	0.34	0.20	0.17	0.30	0.17	0.52	0.30	0.22	0.37	0.66	0.19	0.22	0.38	0.21	0.22	0.24	0.39	0.07	0.41	0.19	0.08
Denmark	1.38	1.28	2.21	1.59	1.90	2.06	1.17	1.41	1.48	0.65	1.07	1.04	1.63	0.84	1.03	1.17	1.37	1.20	1.52	0.77	0.81	1.04	
Dominica	0.00	11.77	0.00	0.00	0.00	0.00	0.00	0.00	0.00	0.00	0.00	0.00	0.00	0.00	0.00	0.00	0.00	0.00	0.00	0.00			
Ecuador	0.36	0.36	0.42	0.63	0.00	0.09	0.53	0.52	0.56	0.65	0.61	0.32	0.82	0.81	1.28	1.11	1.45	0.87	1.37	1.42	0.90	1.03	
Estonia	2.21	0.58	0.00	0.00	0.00	1.09	0.00	0.99	1.55	0.00	0.00	0.00	0.60	0.96	2.01	0.74	1.85	0.23	0.22	0.70	2.20	1.12	
Finland	1.29	1.04	0.84	0.21	0.75	1.20	0.83	0.84	0.72	0.97	0.96	0.71	1.09	0.73	1.50	0.66	1.05	1.35	0.74	0.67	1.07	1.43	
France	1.42	1.31	1.16	1.03	1.26	1.07	1.17	1.24	1.15	1.24	1.37	1.44	1.31	1.43	1.60	1.35	1.41	1.50	1.48	1.29	1.57	1.32	
Georgia	0.00			0.00	0.00	0.00	0.00		0.00	0.20	0.32	0.19	0.34	0.33	0.18	0.56	0.00	0.43	0.00	0.18	0.16		0.00
Germany	0.78	0.67	0.64	0.77	0.64	0.76	0.73	0.87	0.77	0.91	0.97	0.95	0.82	0.85	0.94	1.10	1.13	1.08	1.21	1.11	0.88	0.83	
Grenada	0.00	0.00	0.00	0.00	11.02	0.00	0.00	0.00	0.00	15.45	0.00	0.00	10.29	0.00	0.00	0.00	0.00	0.00	0.00	0.00	0.00		
Guatemala					0.36	0.41	0.39	0.37	0.44	0.12	0.87	0.42	0.65	0.89	0.59	0.80	0.93	0.87	0.92	0.42	0.96	0.82	
Guyana	0.00	0.00	0.00	0.00	0.00	2.83	7.17	3.24	0.00	0.00	1.05	0.00	0.00	0.00	1.41	0.00	0.00	1.50	0.00				
Hungary	0.55	0.98	0.57	1.26	0.74	1.02	1.21	0.76	1.26	0.83	0.76	0.94	0.68	0.97	0.85	0.75	0.80	0.47	0.63	1.02	1.03	0.98	1.19
Iceland	0.00	0.00	2.15	1.80	0.00	5.68	0.00	0.00	0.00	0.00	0.00	7.04	0.00	4.70	2.05	0.00	1.90	0.00	0.00	1.58	3.91	0.00	
Ireland							1.76	2.34	1.84	2.18	1.30	2.62	3.23	1.01	0.69	1.97	2.14	2.65	1.36	1.74	2.07	2.56	
Israel	1.67	2.32	1.63	1.49	1.56	1.42	1.41	2.10	3.20	2.63	2.78	3.56	1.95	3.50	2.25	2.34	1.67	2.26	1.64	1.98	2.00	2.23	
Italy			1.94	1.82	1.98	1.68	1.88	1.90	1.82	1.71	1.64	1.45	1.57	1.38	1.67	1.67	1.66	1.71	2.18	1.93	2.19		
Jamaica	0.92	1.02	0.80	0.77	0.00	1.30			1.36	1.59	1.06	1.87	2.44	0.68									
Japan	1.04	1.15	1.09	1.11	1.05	1.10	0.96	1.12	1.21	1.19	1.12	1.16	1.15	1.16	1.17	1.16	1.24	1.41	1.28	1.52	1.44		
Kuwait	0.40	0.77	0.00	0.00	0.00	0.00	0.00	0.00	0.22	0.00	1.14	0.00	0.00	0.00	0.00	0.00	0.00	0.00	0.16			0.00	0.11
Kyrgyzstan	1.03	0.41	1.28	1.06	1.25	0.26	1.25	0.35	0.20	0.45	0.00	0.75	1.32	0.51	0.36	0.54	0.19	0.99	0.66				
Latvia	1.12	1.64	1.11	0.00	0.30	1.06	0.94	0.43	1.11	0.96	1.08	0.00	1.12	0.18	0.23	0.66	1.16	0.41	0.68	0.55	0.35	0.78	0.93
Lithuania	0.80	0.55	1.67	0.61	0.45	0.96	0.28	0.48	0.68	1.29	1.24	1.54	0.66	0.29	1.30	0.90	0.77	1.57	1.27	1.02	1.58	0.43	0.46
Luxembourg	0.00	1.61	1.86	1.06	1.02	1.01	2.64	0.00	0.00	0.00	2.02	2.65	0.00	3.26	0.00	0.96	0.00	3.00	1.31	0.51	0.50	0.00	
Malta	0.00	0.00	1.53	1.47	2.85	3.40	2.89	3.27	1.34	1.12	1.30	1.15	1.00	1.00	2.75	0.00	4.24	0.81	3.37	0.00	0.00		
Mauritius					0.00	0.00	0.72	0.84	0.00	0.00	0.00	0.84	0.00	0.00	2.08	0.78	0.56	0.00	0.60	0.50	0.00	1.38	1.38
Mexico	1.54	1.15	1.46	1.39	1.46	1.61	1.72	2.29	1.85	1.68	1.95	1.81	1.83	2.01	1.65	2.28	1.80	2.07	2.08	1.82	1.92	2.21	
Netherlands	1.40	1.45	1.43	1.00	0.84	1.35	1.12	0.96	1.34	1.40	1.24	1.40	2.05	1.26	1.76	1.78	1.56	1.46	1.73	1.16	1.24	1.38	1.25
New Zealand	1.95	1.58	1.98	2.26	1.93	2.74	2.46	2.12	2.50	2.77	3.27	3.85	3.12	2.47	2.49	1.90	2.11	2.27					

Nicaragua	0.68	2.59	1.93	2.20	0.20	0.77	0.16	1.22	0.53	0.32	1.91	0.26	1.97	0.00	0.46	1.51	1.16	1.06	1.47	0.62	0.42	0.59	
Norway	1.52	2.01	0.69	0.20	1.01	0.61	0.47	1.53	1.07	0.73	1.82	0.73	0.92	0.84	1.87	1.48							
Panama	0.81	0.65	0.45	3.05	2.31	0.41	3.25	2.41	0.97	0.69	1.48	1.60	0.32	1.12	2.42	0.00	0.79	0.99	0.47		0.45	1.51	
Paraguay	1.74	0.34	0.88	1.56	1.27	1.40	1.62	2.18	2.39	3.03	2.07	2.72	3.49	2.36	3.35	2.21	2.27	2.07	3.65	0.92	1.30		
Philippines	0.16	0.27	0.41			0.14	0.09	0.18	0.14	0.16	0.09			0.12		0.15	0.32	0.40	0.40				
Poland	0.97	0.75	0.59	0.73	0.73	0.90	0.89	0.85	0.74	0.64	0.79	0.77	0.86	0.79	0.81	0.63	0.69	0.84	0.73	0.69	0.86	0.74	
Portugal		0.75	0.79				1.11	0.78	1.05	0.68	0.49	1.14	1.16	1.54	0.90	0.80	1.19	0.89	1.40			0.89	
Republic of Korea	0.10	0.20	0.23	0.42	0.57	0.48	0.42	0.34	0.50	0.46	0.47	0.27	0.60	0.59	0.44	0.48	0.36	0.44	0.44	0.32	0.34	0.44	
Republic of Moldova	0.95	1.20	1.28	1.05	1.02	1.37	1.47	1.18	0.44	0.42	0.20	1.58	0.17	0.75	0.48	0.00	0.59	0.38			0.17		
Romania	0.12	0.17	0.26	0.04	0.40	0.03	0.37	0.20	0.14	0.21	0.22	0.16	0.14	0.12	0.19	0.14	0.06	0.45	0.14				
Saint Kitts and Nevis	0.00	0.00	0.00	0.00	0.00	0.00	0.00	0.00	0.00	17.12	0.00	0.00	0.00	0.00	0.00	0.00							
Saint Lucia	6.20	0.00	0.00	0.00	0.00	0.00		0.00	0.00	0.00	0.00	0.00	0.00	0.00	5.43	0.00	0.00	0.00	4.85	0.00			
Saint Vincent and Grenadines	0.00	0.00	0.00	0.00	0.00	0.00	0.00	9.68	0.00	0.00	0.00	10.44	0.00	0.00	9.31	0.00	0.00	0.00	0.00	0.00	0.00		
Serbia	0.47	0.31	0.70	0.44	0.98	0.48	0.87	0.81	0.94	1.11	0.93	0.27	0.86	0.61	0.86	0.73	0.41	1.38	0.85	1.44	0.47	0.66	0.65
Singapore												0.00	0.15	0.30	0.45	0.34	0.15	0.69	0.66	0.81	0.38	0.51	
Slovakia	0.28	0.34	0.45	0.15	0.75	0.26	0.00	0.68	0.45	0.59		0.64	1.11	1.02		0.52	0.71	0.45	0.65	0.24	0.45	0.17	0.47
Slovenia	1.19	1.73	1.40	0.64	0.60	0.71	0.82	1.91	1.86	2.91	1.96	2.40	1.24	2.61	0.94	1.97	1.47	1.87	0.68	1.27			
South Africa	1.48	0.86	1.23	0.98	0.88	1.06	1.29	1.22	1.08	1.27	1.33	1.27	1.18	1.22	1.20	1.36	1.48	1.39	1.33	0.78			
Spain	0.90	0.85	0.94	0.94	1.12	0.97	0.88	0.97	1.09	1.04	1.09	0.93	1.34	1.19	1.01	1.30	1.27	1.29	1.09	1.35	1.17	1.19	
Suriname	3.01	0.00	4.90	0.00	0.00	0.00	0.00	5.13	0.00	2.48	0.00	3.43	0.00	0.00									
Sweden	1.29	1.48	0.87	1.40	1.87	0.89	1.29	1.21	1.24	0.74	1.59	1.20	1.02	1.32	1.20	1.53	1.48	1.00	1.18	1.28	1.08	0.97	1.39
Switzerland	1.51	2.05	1.80	1.98	0.95	1.86	1.54	1.05	1.73	1.86	1.44	1.63	1.52	0.83	1.44	1.11	1.06	1.48	1.30	0.93	1.43	1.08	
Thailand		1.44	0.87	1.52	0.86	1.03	1.10	1.19	0.89	0.99	1.18	1.06	1.62	1.68	2.07	3.44	3.18	3.76	3.96		2.72		
Turkey									0.81	0.61	0.86	0.80	0.74	0.70	0.49	0.53	0.58	0.56	0.43	0.35	0.34	0.33	0.20
United Kingdom	1.40	1.69	1.51	1.79	1.50	1.51	1.36	1.75	1.68	1.46	1.53	1.67	1.74	1.84	1.79	1.72	1.75	1.86	1.50	1.56	1.36		
United States of America	3.76	3.57	3.54	3.38	3.53	3.24	3.25	3.07	3.08	2.79	2.78	2.70	2.81	2.58	2.38	2.32	2.20	2.09	2.08	1.90	1.83	1.81	
Uruguay	2.23	2.58	0.44	1.56	1.53	2.69	2.12	1.74	1.04	0.93		0.99	1.40	1.09	1.71	2.28	3.87	1.60	1.51	1.29	1.04	2.39	
Uzbekistan				0.00	0.04				0.04	0.00	0.00	0.00	0.08	0.13	0.06	0.27	0.29	0.27	0.25				
Venezuela	1.80	2.09	1.92	2.15	2.10	1.97	2.12	1.91	1.32	1.82	1.15	1.22	1.58	1.39	1.20	1.75							

SSc-ASMR are expressed as deaths per 1,000,000 population. SSc-ASMR = systemic sclerosis age-standardized mortality rate;

Table S7: SSc-ASMR of male and female population across 74 countries

Male

Year	2001	2002	2003	2004	2005	2006	2007	2008	2009	2010	2011	2012	2013	2014	2015	2016	2017	2018	2019	2020	2021	2022	2023
LOESS smoothed rate	0.75	0.73	0.71	0.70	0.68	0.67	0.66	0.65	0.64	0.64	0.65	0.66	0.67	0.68	0.69	0.70	0.70	0.69	0.69	0.68	0.66	0.64	0.62
Upper 95% CI	0.83	0.79	0.76	0.74	0.72	0.71	0.70	0.69	0.68	0.68	0.69	0.70	0.71	0.72	0.73	0.74	0.74	0.73	0.73	0.72	0.72	0.72	0.73
Lower 95% CI	0.66	0.67	0.67	0.65	0.64	0.63	0.61	0.60	0.59	0.60	0.60	0.61	0.62	0.64	0.65	0.66	0.66	0.66	0.65	0.63	0.60	0.56	0.51

Female

Year	2001	2002	2003	2004	2005	2006	2007	2008	2009	2010	2011	2012	2013	2014	2015	2016	2017	2018	2019	2020	2021	2022	2023
LOESS smoothed rate	2.30	2.29	2.28	2.27	2.25	2.24	2.22	2.19	2.17	2.15	2.14	2.14	2.14	2.14	2.16	2.16	2.14	2.12	2.09	2.05	2.00	1.94	1.87
Upper 95% CI	2.54	2.47	2.42	2.39	2.38	2.36	2.34	2.32	2.30	2.28	2.27	2.26	2.27	2.27	2.28	2.28	2.26	2.23	2.21	2.18	2.17	2.18	2.19
Lower 95% CI	2.06	2.12	2.15	2.15	2.13	2.11	2.09	2.06	2.04	2.03	2.01	2.01	2.01	2.02	2.04	2.04	2.03	2.01	1.98	1.92	1.83	1.71	1.56

Data are number of deaths per 1,000,000 population. SSc-ASMR = systemic sclerosis age-standardized mortality rate; LOESS = locally weighted regression; CI = confidence interval

Table S8: SSc-ASMR across 74 countries sorted by regions

Europe

Year	2001	2002	2003	2004	2005	2006	2007	2008	2009	2010	2011	2012	2013	2014	2015	2016	2017	2018	2019	2020	2021	2022	2023
LOESS smoothed rate	0.99	1.00	1.01	1.02	1.03	1.04	1.04	1.05	1.05	1.05	1.05	1.05	1.05	1.06	1.08	1.09	1.09	1.08	1.06	1.03	0.98	0.92	0.84
Upper 95% CI	1.10	1.08	1.07	1.07	1.08	1.09	1.10	1.10	1.11	1.11	1.11	1.10	1.10	1.11	1.13	1.14	1.14	1.13	1.11	1.08	1.04	1.00	0.96
Lower 95% CI	0.89	0.93	0.95	0.97	0.98	0.99	0.99	1.00	1.00	1.00	1.00	1.00	1.00	1.01	1.03	1.04	1.04	1.04	1.02	0.98	0.91	0.83	0.72

Americas

Year	2001	2002	2003	2004	2005	2006	2007	2008	2009	2010	2011	2012	2013	2014	2015	2016	2017	2018	2019	2020	2021	2022	2023
LOESS smoothed rate	2.16	2.15	2.13	2.11	2.08	2.05	2.02	1.99	1.95	1.92	1.89	1.87	1.85	1.84	1.83	1.80	1.76	1.73	1.70	1.66	1.63	1.59	NA
Upper 95% CI	2.37	2.30	2.25	2.21	2.19	2.16	2.13	2.10	2.06	2.03	2.00	1.98	1.96	1.95	1.93	1.90	1.86	1.83	1.79	1.78	1.78	1.79	NA
Lower 95% CI	1.96	2.00	2.02	2.01	1.98	1.95	1.91	1.87	1.84	1.80	1.78	1.76	1.75	1.74	1.72	1.69	1.66	1.63	1.60	1.55	1.48	1.39	NA

Western Pacific

Year	2001	2002	2003	2004	2005	2006	2007	2008	2009	2010	2011	2012	2013	2014	2015	2016	2017	2018	2019	2020	2021	2022	2023
LOESS smoothed rate	0.86	0.86	0.86	0.86	0.86	0.86	0.87	0.86	0.87	0.91	0.95	0.97	0.97	0.97	0.95	0.94	0.96	1.01	1.08	1.18	1.31	1.46	1.64
Upper 95% CI	1.17	1.09	1.04	1.02	1.02	1.03	1.04	1.04	1.05	1.09	1.14	1.14	1.15	1.16	1.13	1.12	1.13	1.17	1.25	1.39	1.59	1.86	2.17
Lower 95% CI	0.56	0.63	0.68	0.70	0.70	0.70	0.69	0.69	0.69	0.74	0.77	0.79	0.79	0.78	0.77	0.76	0.79	0.85	0.91	0.97	1.02	1.07	1.11

Data are number of deaths per 1,000,000 population. SSc-ASMR = systemic sclerosis age-standardized mortality rate; LOESS = locally weighted regression; CI = confidence interval; NA = not available.

Table S9: SSc-ASMR across 74 countries sorted by SDI group

High SDI

Year	2001	2002	2003	2004	2005	2006	2007	2008	2009	2010	2011	2012	2013	2014	2015	2016	2017	2018	2019	2020	2021	2022	2023
**LOESS smoothed rate**	2.05	2.02	1.98	1.95	1.92	1.90	1.88	1.86	1.84	1.82	1.80	1.78	1.76	1.73	1.70	1.67	1.64	1.60	1.56	1.52	1.48	1.44	1.39
**Upper 95% CI**	2.30	2.20	2.13	2.08	2.05	2.03	2.01	2.00	1.97	1.96	1.95	1.92	1.89	1.87	1.84	1.81	1.77	1.73	1.69	1.66	1.66	1.68	1.72
**Lower 95% CI**	1.80	1.83	1.84	1.82	1.80	1.76	1.74	1.72	1.70	1.69	1.66	1.64	1.62	1.59	1.57	1.54	1.51	1.48	1.44	1.38	1.30	1.19	1.06

High-middle SDI

Year	2001	2002	2003	2004	2005	2006	2007	2008	2009	2010	2011	2012	2013	2014	2015	2016	2017	2018	2019	2020	2021	2022	2023
**LOESS smoothed rate**	0.99	1.09	1.17	1.22	1.25	1.26	1.25	1.21	1.17	1.14	1.12	1.10	1.09	1.09	1.10	1.11	1.10	1.08	1.05	1.01	0.95	0.88	0.80
**Upper 95% CI**	1.25	1.28	1.31	1.34	1.36	1.38	1.36	1.32	1.29	1.26	1.23	1.21	1.20	1.20	1.20	1.21	1.20	1.18	1.15	1.11	1.08	1.05	1.03
**Lower 95% CI**	0.74	0.91	1.03	1.10	1.13	1.14	1.13	1.09	1.05	1.03	1.01	1.00	0.98	0.98	0.99	1.00	1.00	0.99	0.96	0.90	0.82	0.70	0.56

Middle SDI

Year	2001	2002	2003	2004	2005	2006	2007	2008	2009	2010	2011	2012	2013	2014	2015	2016	2017	2018	2019	2020	2021	2022	2023
**LOESS smoothed rate**	0.98	0.99	1.01	1.02	1.04	1.04	1.05	1.06	1.07	1.09	1.13	1.17	1.23	1.30	1.36	1.40	1.44	1.47	1.50	1.53	1.56	1.58	NA
**Upper 95% CI**	1.23	1.18	1.15	1.15	1.16	1.17	1.18	1.20	1.20	1.23	1.26	1.31	1.36	1.43	1.49	1.53	1.56	1.59	1.63	1.69	1.77	1.88	NA
**Lower 95% CI**	0.72	0.81	0.87	0.90	0.91	0.92	0.92	0.92	0.94	0.96	1.00	1.04	1.10	1.18	1.23	1.28	1.32	1.35	1.37	1.37	1.34	1.28	NA

Data are number of deaths per 1,000,000 population. SSc-ASMR = systemic sclerosis age-standardized mortality rate; SDI = sociodemographic index; LOESS = locally weighted regression; CI = confidence interval; NA = not available.

Table S10: AAPC in SSc-ASMR, 2010-2023

	Country	Period	AAPC	[95%CI]		P-value
1	Argentina	2010-2022	-1.42	-4.96	2.23	0.3979
2	Australia	2010-2023	-0.81	-1.94	0.37	0.1768
3	Austria	2010-2023	-3.48	-8.89	1.88	0.1836
4	Belgium	2010-2021	1.10	-2.44	4.92	0.5075
5	Brazil	2010-2021	-0.51	-1.54	0.64	0.3575
6	Canada	2010-2022	-0.89	-2.55	0.83	0.3131
7	Chile	2010-2021	0.62	-4.20	5.84	0.7299
8	Colombia	2010-2021	0.77	-1.19	3.04	0.2939

9	Costa Rica	2010-2022	0.09	-4.03	4.94	0.8654
10	Croatia	2010-2021	-3.86	-14.77	7.36	0.4095
11	Cuba	2010-2021	0.33	-5.59	6.63	0.9086
12	Czech Republic	2010-2023	-4.98	-14.32	4.19	0.2452
13	Denmark	2010-2022	0.34	-5.79	7.35	0.8302
14	Ecuador	2010-2022	5.85*	0.14	15.07	0.0468
15	Finland	2010-2022	1.55	-6.33	9.75	0.5939
16	France	2010-2022	0.49	-1.08	2.17	0.4839
17	Germany	2010-2022	-1.45	-4.09	1.19	0.1544
18	Guatemala	2010-2022	2.81	-1.87	9.13	0.2260
19	Hungary	2010-2023	2.30	-1.25	5.79	0.1960
20	Ireland	2010-2022	0.00	-6.93	8.14	0.9094
21	Israel	2010-2022	-3.80*	-7.29	-0.38	0.0316
22	Italy	2010-2021	2.40*	0.60	5.00	0.0084
23	Japan	2010-2021	2.31*	1.21	3.59	< 0.001
24	Lithuania	2010-2023	-8.37	-18.28	2.65	0.0928
25	Mexico	2010-2022	1.16	-0.88	3.41	0.2112
26	Netherlands	2010-2023	0.14	-5.13	5.03	0.9910
27	New Zealand	2010-2018	-4.79*	-9.07	-1.34	0.0068
28	Norway	2010-2016	6.36	-10.76	30.26	0.4399
29	Paraguay	2010-2021	-8.44	-19.51	5.08	0.1116
30	Philippines	2010-2019	14.08*	5.54	35.81	0.0032
31	Poland	2010-2022	0.10	-2.18	2.51	0.8858
32	Portugal	2010-2022	1.58	-8.99	13.47	0.5435
33	Republic of Korea	2010-2022	-2.29	-6.01	1.45	0.2400
34	Romania	2010-2019	3.36	-9.47	18.39	0.5559
35	Serbia	2010-2023	-0.31	-6.50	6.14	0.9078
36	Slovakia	2010-2023	-7.12	-17.11	2.15	0.1100
37	Slovenia	2010-2020	-7.54	-16.36	0.98	0.0796
38	South Africa	2010-2020	-3.01	-7.19	3.00	0.1592
39	Spain	2010-2022	1.20	-1.29	4.02	0.2691

40	Sweden	2010-2023	-0.08	-4.18	4.20	0.9846
41	Switzerland	2010-2022	-2.91	-6.43	0.56	0.0988
42	Thailand	2010-2021	11.04*	7.59	16.73	< 0.001
43	Turkey	2010-2023	-6.66*	-10.51	-4.44	< 0.001
44	United Kingdom	2010-2021	-0.65	-1.64	0.42	0.2284
45	United States of America	2010-2022	-3.79*	-4.34	-3.30	< 0.001
46	Uruguay	2010-2022	10.22*	1.54	22.14	0.0248
47	Venezuela	2010-2016	0.27	-11.94	15.60	0.9074

*AAPC was considered statistically significant if $p < 0.05$ and the 95% CI did not include zero;
SSc = systemic sclerosis; AAPC = average annual percent changes; CI = confidence interval
